# Disinfection of aircraft: Appropriate disinfectants and standard operating procedures for highly infectious diseases

**DOI:** 10.1007/s00103-016-2460-2

**Published:** 2016-10-26

**Authors:** Joachim Klaus, Peter Gnirs, Sabine Hölterhoff, Angela Wirtz, Matthias Jeglitza, Walter Gaber, Rene Gottschalk

**Affiliations:** 1 Occupational Safety Flight Operations, Department FRA PX/O, Lufthansa Basis – Tor 21, Lufthansa German Airlines, 60546 Frankfurt am Main, Germany; 2 Lufthansa Technik AG, Frankfurt am Main, Germany; 3 Hesse Ministry of Social Affairs and Integration, Wiesbaden, Germany; 4 Federal Ministry of Transport and Digital Infrastructure, Berlin, Germany; 5 Fraport AG Frankfurt Airport Services Worldwide, Frankfurt am Main, Germany; 6 Health Protection Authority and Competence Network for Highly Pathogenic Agents, Frankfurt am Main, Germany

**Keywords:** Air traffic, Highly infectious diseases, Disinfection of aircraft, Standardized operating procedures, Substances for disinfection, Luftverkehr, Hochansteckende Krankheiten, Desinfektion von Flugzeugen, Standardverfahren, Produkte zur Desinfektion

## Abstract

For infectious diseases caused by highly pathogenic agents (e. g., Ebola/Lassa fever virus, SARS-/MERS-CoV, pandemic influenza virus) which have the potential to spread over several continents within only a few days, international Health Protection Authorities have taken appropriate measures to limit the consequences of a possible spread. A crucial point in this context is the disinfection of an aircraft that had a passenger on board who is suspected of being infected with one of the mentioned diseases. Although, basic advice on hygiene and sanitation on board an aircraft is given by the World Health Organization, these guidelines lack details on available and effective substances as well as standardized operating procedures (SOP). The purpose of this paper is to give guidance on the choice of substances that were tested by a laboratory of Lufthansa Technik and found compatible with aircraft components, as well as to describe procedures which ensure a safe and efficient disinfection of civil aircrafts. This guidance and the additional SOPs are made public and are available as mentioned in this paper.

## Zusammenfassung

Weltweit haben Gesundheitsbehörden geeignete Maßnahmen getroffen, um die Verbreitung infektiöser Krankheiten zu begrenzen. Erkrankungen, die durch hochpathogene Erreger (wie z. B. Ebola-/Lassa-Fieber-Viren, SARS-/MERS-CoV, pandemische Grippeviren) ausgelöst werden, haben das Potenzial, sich innerhalb weniger Tage auf mehreren Kontinenten zu verbreiten. In diesem Zusammenhang ist die zügige und qualifizierte Desinfektion eines Verkehrsflugzeugs, das einen (möglicherweise) infizierten Passagier an Bord hat, ein entscheidender Punkt. Trotz vorhandener grundlegender Richtlinien seitens der Weltgesundheitsorganisation (WHO) zur Hygiene an Bord von Flugzeugen fehlt es an detaillierten Hinweisen zu Desinfektionsstandards und zu verwendenden Produkten, die sowohl in der Wirksamkeit, aber auch im Sinne der Materialverträglichkeit für Flugzeugkomponenten unbedingt notwendig sind. Diese Abhandlung soll zeigen, wie mit einer Auswahl an Produkten, die für Flugzeugteile materialverträglich sind, mittels festgelegter Anwendungstechniken eine sichere und effiziente Desinfektion von zivilen Flugzeugen gewährleistet werden kann. Diese Standardverfahren werden der Öffentlichkeit im Sinne einer Leitlinie zur Verfügung gestellt.

## Introduction

Since there are flight connections to nearly all regions of the world, Health Protection Authorities of all countries must focus on the problem of epidemic spread via civil aviation. Fortunately, it does not happen often that passengers become infected by contagious co-passengers [1]. Nevertheless, we have to expect that contamination of an aircraft through infected passengers is a realistic scenario. To date there are no mandatory guidelines from WHO for disinfection of aircrafts in case of highly pathogenic agents.

**Fig. 1:**
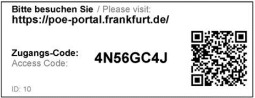
Link for the complete set of High Infectious Diseases Forms (HID Forms) on Lufthansa aircraft from Lufthansa Technik AG. The forms may be used for your own purposes. Lufthansa would appreciate a short feedback (e-mail FRAPXO@DLH.DE) if you use the SOPs

In recent cases of infectious diseases caused by highly pathogenic agents (e. g., Ebola fever virus, Lassa fever virus, SARS-CoV, MERS-CoV, pandemic influenza virus) which have the potential to spread over several continents within only a few days, international health protection authorities took measures – which are, in part, of high economic relevance – to limit the consequences of a possible spread [1–8]. The measures taken did not in all cases reach the intended objective or were more or less inefficient considering the effected expenditure [1, 6]. Therefore, it is necessary to examine mechanisms and relevance of such transmissions in relation to international public health. The results will enable us to deduce whether measures must be taken and which ones would be necessary to limit the spread of various, highly pathogenic and life-threatening infectious diseases [5, 10–13]. If passengers infected with diseases that have the potential to become a public health emergency of international concern use public transportation (i.e., aircrafts), there is a potential threat to the destination area and – depending on the route of infection – for passengers travelling with the same aircraft [1, 3, 5, 10, 13].

Based on the framework of the International Health Regulations, a number of recommendations, standard procedures, and guidelines focusing on the operational needs of civil aviation have been published. Basic advice on hygiene and sanitation on board an aircraft is specified by the “Guide to Hygiene and Sanitation in Aviation”, published by the WHO [9]. Nevertheless, the WHO guidelines lack details on available and effective substances as well as standardized operating procedures (SOP). In addition, national legislation or manufacturers’ guidance is lacking.

Every aircraft undergoes cleaning based on a standard cleaning procedure (SOP) prior to the next departure. In case of a suspicious or confirmed passenger suffering a highly infectious disease (HID), a special, additional disinfection of the aircraft is mandatory. For this purpose, all used disinfectants must be aircraft component compatible, i. e., must not have any negative effects on individual parts or the structure of the aircraft, while also fulfilling national healthcare requirements [13, 14]. When choosing a disinfectant, it must be ascertained that their application will cause neither short- nor long-term damage to

- the aircraft structure (i.e., corrosion),
- electronics and avionics (i.e., insulation of cables),
- sensors (i.e., smoke detection),
- interior (i.e., installations, seats, monitors, media devices, windows, galleys, countertops, restrooms).

To this day and considering national and international law and regulations, sufficient recommendations from aircraft manufacturers and WHO are not available [15]. Their references mainly focus on aircraft component compatibility and not on ensuring safe disinfection of surfaces or official requirements. International airlines have to be able to use a product which is recognized worldwide. The most efficient disinfection of aircrafts has to be guaranteed without endangering the

- safety of passengers and crew,
- operability of aircrafts,
- aircraft installations, and
- aircraft certifications.

Apart from obvious aspects of health, internationally accepted requirements and specifications of authorities, the financial aspects of airlines must be taken into consideration. Concerns are

- delays and rebookings,
- flight cancellations and additional accommodation of passengers,
- ferry flights, and
- storage/transport of material and equipment for disinfection (dangerous goods).

## Objective

The purpose of this paper is to fill in these gaps and to give guidance on a selection of substances that were tested and found compatible with aircraft components, as well as to describe procedures that ensure a safe and efficient disinfection of civil aircrafts.

This guidance and these additional standard operating procedures (SOP) are made public and are available as indicated in the references [14] or via QR code/link as shown in Fig. 1.

**Fig. 2:**
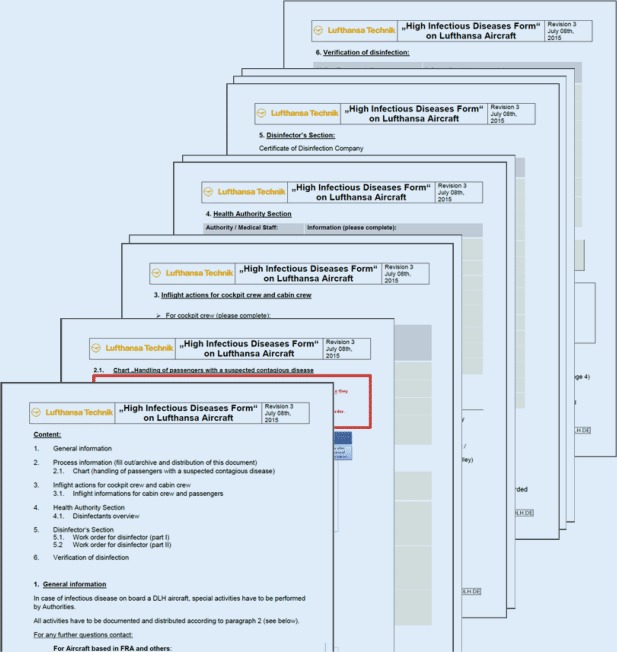
Standard operating procedures from Lufthansa Technik to be used in aircrafts for disinfection in the presence of high infectious diseases

In 2014, the Lufthansa Group (Lufthansa German Airlines, Austrian, Swiss, Lufthansa Cargo and others) operated a worldwide network with 217 destinations in 107 countries and carried out over one million flights, transporting nearly 106 million passengers. Lufthansa considers it as absolutely necessary to implement the SOPs in accordance with local Health Protection Authorities to ensure international air traffic without interruption. The airline will apply these SOPs worldwide and would like them to be accepted by all sides (Fig. 2).

Since expert attention to incidents and accurate procedures vary greatly in different countries, Lufthansa implemented specific SOPs and information which comply with guidelines of authorities and airline interests as well as the International Air Transport Association (IATA). These SOPs include procedures for

- cockpit crew,
- cabin crew,
- passengers on board aircrafts,
- on board, ground, authorities communication,
- local Health Protection Authorities,
- disinfectors,
- maintenance and handling personnel,
- airport partners,

and shall provide the safe and hygienic operation of the aircraft.

The process should be kept as simple and cost efficient as possible for all concerned parties, such as local authorities, affected airlines, and airports. The goal is to reach a conclusive, internationally accepted medical and legislative procedure for disinfection which enables a safe, rapid, and cost effective further utilization in case of a passenger with a highly infectious disease on board any civil aircraft.

The authors took these critical topics into consideration and worked closely with local Health Protection Authorities (HPA) as well as colleagues from the Frankfurt International Airport (FRAPORT AG) while testing different disinfectants and techniques before implementing them.

## Materials and methods

Lufthansa Technik AG (LHT) is a wholly owned subsidiary of Lufthansa German Airlines. It is an internationally licensed maintenance, production, and development organization and is an authorized design organization (design organization approval certificate EASA.21.J.019). Therefore, LHT is authorized to test the use of disinfectants and related procedures in aircraft interiors based on international, standardized testing methods. In Lufthansa Technik Central Laboratories, a comprehensive evaluation was performed to confirm the compatibility of aviation materials with substances and agents for surface treatment and cleaning.

With a broad variety of tailored LHT evaluation programs, producers, suppliers, and users can check, prove, and substantiate that subject agents do not cause damage or compromise the airworthiness of the aircraft and its components. For the disinfection process several products have been determined as useful according to officially nominated disinfection methods [12]. Thus, Lufthansa Technik Central Laboratories tested several agents regarding harmful effects when applied to specific aviation materials and in compliance with aircraft specifications such as Boeing D6-17487, Evaluation of Airplane Maintenance Materials, or Airbus AIMS 09-00-002, Evaluation of Maintenance Materials.

The products taken into service are belonging to to the family of alcohol-based agents, formaldehyde-based agents, and oxygen-releasing disinfectants. The use of formaldehyde-based agents reveals an increased ablation of magnesium, which shows that it can be used for single purposes, but not for regular interval cleaning. Based on the testing, the group of oxygen-releasing disinfectants shall not be used on surfaces with magnesium due to a high risk of material corrosion and on seals because of the possibility of embrittlement and consecutive malfunction. Alcohol-based disinfectants were tested without any restriction. Nevertheless, it must be noted that alcohol-based agents are flammable and the explosive level has to be closely observed during their use. It is mandatory to refer to the HID Form from Lufthansa Technik [14] or via the QR code/link as shown in Fig. 1.

## Results

Multiple test procedures were used to examine the interdependencies between typical aviation materials and the substances tested. The LHT Central Laboratories provide services to perform the tests in its chemical and metallographic laboratories and also according to specific standards of Lufthansa, Boeing, Airbus, American Standard Methods (ASTM), etc. They tested effects on aircraft materials such as metal, glass, electric conduits, synthetics, leather, and fabric seat covers, windows, and monitors. All disinfectants are approved by the airline engineering and can be used on the Lufthansa fleet. Gathered results deviating from common aircraft manuals, for example, the Aircraft Maintenance Manual (AMM), are documented in the LHT Standard Practices Manual (SPM), which is mandatory for the Lufthansa fleet and, if accepted, for clients.

With regard to the SOPs, different procedures are defined and reduced to simple, reproducible processes [14]. The following procedures were developed and already implemented within the Lufthansa fleet by:

- integration into manuals for cockpit crews
- information
- on notification procedures to air traffic control and operational control center (OCC),
- for cabin crews,
- for passengers,
- for Health Protection Authorities at Port of Destination (POD) or Port of Entry (POE),
- on products and generics,
- for disinfectors,
- for aircraft maintenance and ground personnel, and
- for release to service (RTS).

The above-mentioned procedures are available at Lufthansa destinations worldwide, published in the Station Emergency Reaction Plan (SERP). The SOPs are available in a document on board DLH aircrafts (available for technical/expert groups (see [14] or via QR code/link as shown in Fig. 1)).

## Discussion

Because of a lack of internationally accepted requirements for a suspected or confirmed cause of HID, airlines cannot fall back on coordinated, authorized, and internationally approved procedures. Therefore, the necessity arises to

- discuss the way how infectious diseases are handled on board aircrafts,
- standardize disinfection,
- define “aircraft component compatible” disinfectants and procedures, and
- establish a safe onward flight operation.

To correctly deal with life-threatening diseases caused by highly pathogenic causative agents, the continuous adaptation of procedures to new findings, real-life experiences, and circumstances is absolutely essential. In addition, close coordination with concerned national as well as international Health Protection Authorities must be guaranteed at all times. For example, chloroxylenol (one component of Eco Tru®) was tested and approved by the United States Environmental Protection Agency (EPA) and is authorized for use in the US and Asia, but does not comply with requirements of Health Protection Authorities in Germany [16]. Thus, Lufthansa German Airlines as an internationally operating airline has accepted the challenge and taken first measures in close cooperation with the appropriate Federal and Federal State Ministries, the Health Protection Authority City of Frankfurt and the Fraport Int’l Airport.

Only a small number of disinfectants were chosen to facilitate worldwide transportation and the storage of necessary products with tested component compatibility as well as to reduce the complexity of the matter. Products were selected based on the components

- formaldehyde,
- hydrogen peroxide, and
- alcohol.

These components are effective against HID and they are aircraft component compatible if used properly. In addition, they are available worldwide. Formaldehyde, hydrogen peroxide, and alcohol allow the varying techniques of standard disinfection of surfaces.

Lufthansa Technik, with its certification and expertise, is able to disinfect and to certify the affected aircrafts for release to service effectively and in a time-efficient manner. The past has shown that the negative consequences of missing or uncoordinated procedures could be immense. Abilities and capacities of major airports and involved health protection authorities could be quickly exceeded. Thus, it is highly urgent to define global measures and procedures to ensure that all involved players worldwide coordinate and accept them.

In case of an emergency, being well prepared will ensure optimal processes while minimizing the rate of errors. As a consequence of this, Lufthansa German Airlines developed together with the German competent authorities a High Infectious Diseases (HID) Form which is also available for technical/expert groups via email (FRAPXO@DLH.DE).
